# Improvement of Chemical Composition of *Tisochrysis lutea* Grown Mixotrophically under Nitrogen Depletion towards Biodiesel Production

**DOI:** 10.3390/molecules25204609

**Published:** 2020-10-10

**Authors:** Adel W. Almutairi

**Affiliations:** Biological Sciences Department, Rabigh-Faculty of Science & Arts, King Abdulaziz University, P. O. Box 344, Rabigh 21911, Saudi Arabia; aalmutairi@kau.edu.sa

**Keywords:** protein, microalgae, fatty acids, biodiesel, carbohydrates, pigments

## Abstract

In the present study, the marine microalga *Tisochrysis lutea* was cultivated mixotrophically in F2 growth medium with sodium acetate as exogenous carbon source. The medium was composed of different concentrations of nitrogen to determine the impact of nitrogen depletion on cellular growth and chemical composition. Nitrogen depletion led to severely decreased growth and protein content. However, mild nitrogen depletion (0.22 mM NaNO_3_) led to maximum lipid yield. The fatty acid methyl ester profile also showed increased unsaturation as the nitrogen content decreased. Growth in nitrogen-free medium increased the proportions of mono- and poly-unsaturated fatty acids, while the proportion of saturated fatty acids decreased. Growth under all tested nitrogen levels showed undetectable fatty acids with ≥4 double bonds, indicating these fatty acids had oxidative stability. In addition, all tested nitrogen concentrations led to specific gravity, kinematic viscosity, iodine value, and cetane number that meet the standards for Europe and the U.S.A. However, growth in the presence of nitrogen deficiency enhanced the higher heating value of the resulting biodiesel, a clear advantage from the perspective of energy efficiency. Thus, mixotrophic cultivation of *T. lutea* with nitrogen limitation provides a promising approach to achieve high lipid productivity and production of high-quality biodiesel.

## 1. Introduction

Extensive use of petroleum to produce fuel oil and its derivatives is not sustainable in the long-term because of resource depletion and increasing levels of greenhouse gases [1,2]. In addition, use of fossil fuels contributes to air pollution, and recent research attributed approximately 4.2 million human deaths per year to air pollution [3]. There is therefore increasing attention on research initiatives that are developing renewable, sustainable, and environmentally friendly sources of energy [4,5]. In this context, biodiesel is a widely recognized and promising replacement for fossil fuels whose use may mitigate the effects of greenhouse gas emissions [6]. Usually, biodiesel is produced by transesterification of animal fats or vegetable oils in the presence of a suitable catalyst, resulting in production of glycerol and esters [7]. Many recent studies have focused on biodiesel production from oleaginous microalgae because of their high lipid content, fast growth, and minimal requirements for land. Moreover, because microalgae can be cultivated in non-agricultural land, there is less competition with edible food crops than first generation (food crops) and second generation (agricultural waste) sources of biodiesel [8,9,10,11]. However, production of biodiesel from microalgae is not yet economically feasible [1,9,12].

Current research in this area is focusing on the search for new microalgal strains with high lipid content [13], optimizing production of lipids and biomass [14,15,16], enhancement of lipid production [17,18], reducing the production costs associated with harvesting [19,20], exploiting microalgal by- products [21,22], and reducing the overall costs of microalgal cultivation [23,24]. Some of these goals can be achieved by the introduction of abiotic stress, such as nutrient starvation and alterations in light intensity, temperature, salinity, and pH [2,18,25]. Nitrogen is considered as one of the critical nutrients that significantly affects the biochemical composition and lipid yield of microalgae [26,27,28]. Generally, nitrogen in its different forms plays a vital role in the macromolecular biosynthetic pathways in microalgae to form proteins, amino acids, and chlorophyll [1,29]. Sufficient nitrogen concentration results in a metabolic balance between nitrogen assimilation and the carbon fixation rate [29]. However, nitrogen limitation modifies the cellular carbon flux, in which protein production declines and lipid storage increases [30,31]. Many studies on the effect of nitrogen deficiency on microalgae have been performed under autotrophic conditions. However, nitrogen impact on the main biochemical components of marine microalgae under mixotrophic conditions requires further investigation. Therefore, the present study evaluated the influences of nitrogen limitation on cellular growth and levels of carbohydrates, proteins, pigments, lipids, and fatty acids of the marine haptophyte *Tisochrysis lutea* grown mixotrophically in the presence of sodium acetate. In addition, the suitability of the resulting lipids for biodiesel production and biodiesel characteristics were evaluated.

## 2. Results and Discussion

### 2.1. Effect of Nitrogen Depletion on Growth

#### 2.1.1. Dry Weight

**Figure 1:**
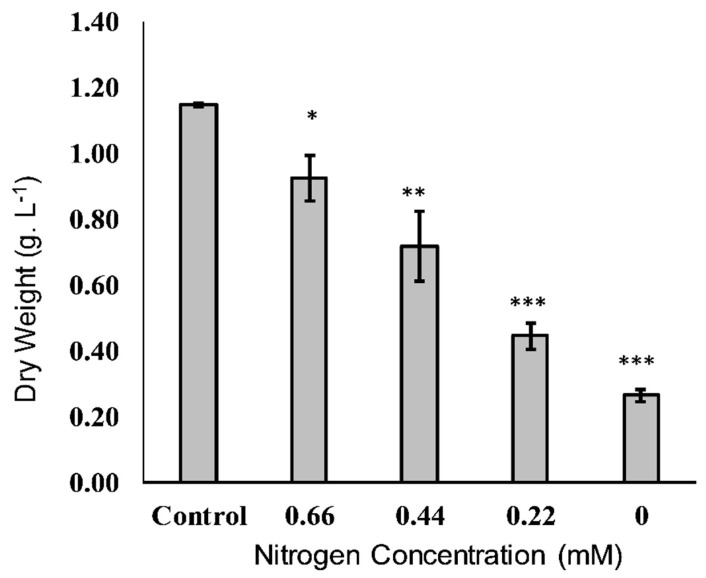
The cellular dry weight of *Tisochrysis lutea* grown mixotrophically at different concentrations of NaNO_3_. * *p* < 0.05, ** *p* < 0.01, *** *p* < 0.001 with respect to the control (0.88 mM).

Dry microalgal biomass typically contains about 50% carbon and 10% nitrogen, and their typical responses to nitrogen deficiency are drastic declines in dry weight (DW) and growth [14]. Under certain stressful conditions, such as nitrogen deficiency, cells tend to survive by accumulating large amounts of carotenoids and lipids [2]. High levels of irradiation, salinity, organic carbon, and free radicals can also induce these responses. Relative to the standard conditions (0.88 mM NaNO_3_), nitrogen depletion in the current study resulted in a linear decline in dry cell weight (Figure 1).

Cellular death during the increased carotenogenesis in the absence of nitrogen can be prevented by enrichment of the algal growth medium with an extra carbon source [32], because the exo-organic carbon (e.g., sodium acetate) seems to function as a substrate for fatty acid and lipid biosynthesis (Section 2.2.4). Thus, the addition of organic carbon increases the lipid content and helps to maintain microalgal growth in the presence of nitrogen depletion. However, carbon supplementation does not stimulate growth as much as nitrogen enrichment. Nitrogen deficiency dramatically reduces the N:P ratio, which in turn severely reduces algal growth. In particular, Rasdi and Qin reported the enhancement of *T. lutea* growth by increasing the N:P ratio from 5:1 to 20:1, but a gradual decline in growth was recorded when the N:P ratio further increased to 120:1 [33]. Similarly, Mayers et al. reported that the lower N:P ratios (16:1 and 32:1) resulted in greater cell density of *Nannochloropsis* sp. than higher ratios of 64:1 and 80:1 [34].

Previous researchers studied the effects of abiotic stress on microalgal growth in efforts to enhance biomass and lipid accumulation [16,25,35]. Different abiotic stressors can lead to changes in physiological and biochemical processes [36] and stimulate production of antioxidants [17], leading to changes in morphology and growth. Thus, commercial-scale studies used different abiotic stressors to stimulate the production of pigments and lipids in microalgae. Alterations in salinity and pH showed a significant impact on the metabolism of lipids and fatty acids in different microalgal species [37,38]. However, the response to nitrogen depletion varies among different species of microalgae. For instance, Yu et al. reported that low concentration of nitrogen (36 mg L^−1^) significantly decreased the growth rate of *Tisochrysis galbana* but slightly increased the growth rate of *Isochrysis zhangjiangens* [5]. Therefore, it is important to examine the impact of different nitrogen concentrations for each species of microalgae.

#### 2.1.2. Growth Rate and Inhibition

The growth rate of *T. lutea* and the inhibitory effect of nitrogen limitation were calculated as listed in (Table 1). The results confirmed the inhibitory effect of nitrogen depletion on growth rate. The lowest growth rate (0.059 d^−1^) was recorded in the nitrogen-free medium, and this growth rate was 68.4% lower than that of the control medium (0.187 d^−1^). These results also indicated a close relationship between nitrogen deficiency and the inhibitory effect. Similarly, Zhu et al. [39] and Pancha et al. [40] concluded that the growth rate and biomass productivity of *Chlorella* sp. and *Scenedesmus* sp. decreased as the nitrogen supply declined. An earlier study reported that a nitrogen concentration of at least 10% of the optimal level is required for optimal production of lipids and carotenoids [41]. This low concentration of nitrogen preserves metabolic function (enzymatic catalysis and hormone production) and reduces protein decomposition. The growth of *T. lutea* in nitrogen limited and nitrogen-free medium might be attributed to the supportive effect of the supplemental carbon (discussed above).

### 2.2. Chemical Composition

#### 2.2.1. Protein

**Figure 2:**
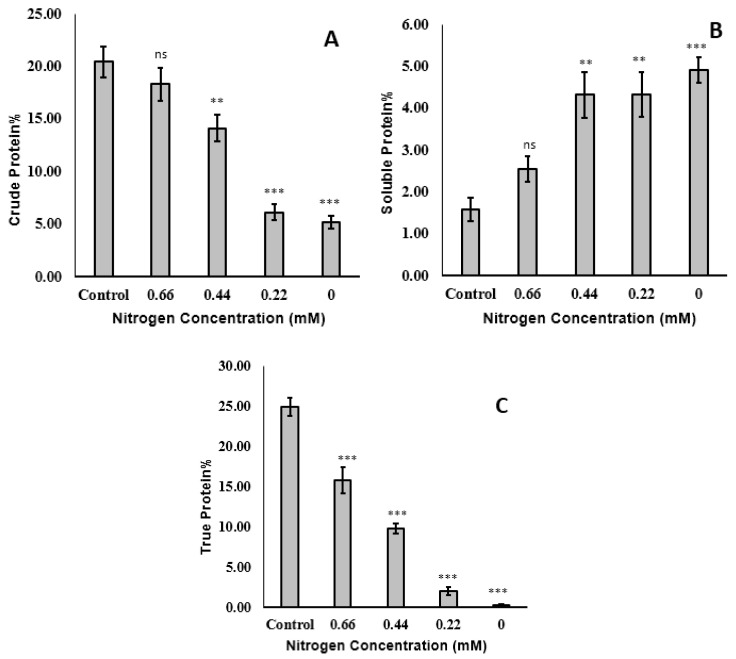
Crude protein (**A**), soluble protein (**B**), and true protein (**C**) content (as % dry weight (DW)) of *Tisochrysis lutea* grown mixotrophically in different concentrations of NaNO_3_. ** *p* < 0.01, *** *p* < 0.001, ns: not significant with respect to the control (0.88 mM).

Nitrogen deficiency also markedly affected the levels of crude, soluble, and true protein in *T. lutea* (Figure 2). In particular, nitrogen deficiency led to protein decomposition, as indicated by reduced levels of crude protein and real protein and an increased level of soluble protein. This drastic protein decomposition can increase the alkalinity of the medium due to the liberation of ammonia, which inhibits microalgal growth at high concentrations [42]. On a DW basis, production of *Tisochrysis lutea* in 3.0 m^3^ outdoor pilot-scale photobioreactors showed a biomass production of up to 20 g m^−2^ day^−1^, with 45% DW proteins and 25% DW lipids [43].

In addition, the increased level of soluble protein in the presence of nitrogen depletion might be ascribed to an increased de novo synthesis or an increase of enzymatic systems in response to the adverse effect of nitrogen deficiency. For instance, increased de novo synthesis of phytol desaturase occurs during conditions that favor carotenogenesis or hyper-accumulation of carotenoids and lipids in different microalgal species. Among the many different nutrients in algal growth medium that affect the total nitrogen and protein content of the algal biomass, nitrogen is considered the most important. Addition of an excess of nitrogen in the growth medium led to increased incorporation of nitrogen into proteins in *Scenedesmus* sp. and increased cellular nitrogen level in *Calcidiscus leptoporus* [44,45]. Growth of *Isochrysis galbana* in the presence of nitrogen depletion during lipid accumulation led to dramatic changes in the levels of 45 of 900 proteins identified in 2-dimensional electrophoresis [46]. These researchers identified 27 of these proteins and reported that they had functions in signal transduction, energy transformation/production, cellular metabolism, transcription/translation, molecular chaperones, defense mechanism, and cytoskeletal structure [46]. Alterations in the levels of these proteins thus reduced cellular growth and photosynthesis, but also increased the accumulation of lipids.

#### 2.2.2. Total Carbohydrates

**Figure 3:**
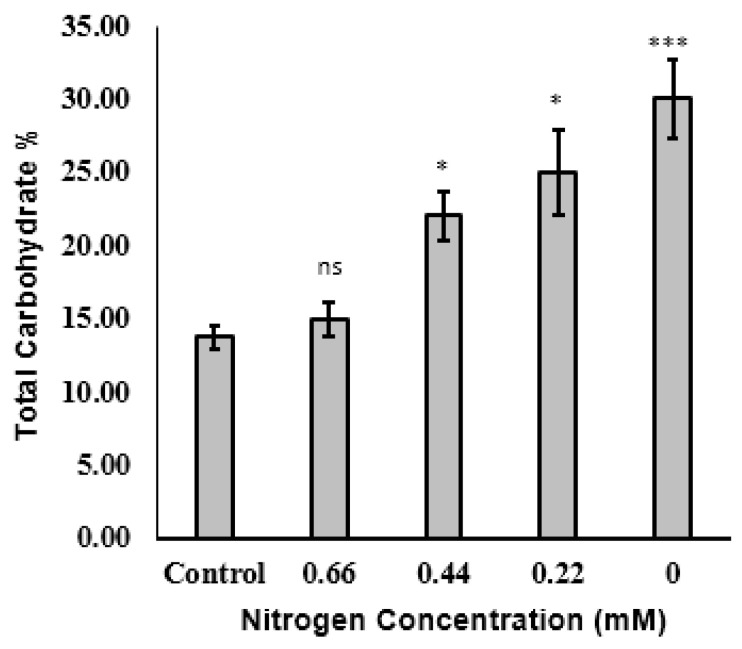
Total carbohydrate content (as % DW) of *Tisochrysis lutea* grown mixotrophically in different concentrations of NaNO_3._ * *p* < 0.05, *** *p* < 0.001, ns: not significant with respect to the control (0.88 mM).

Nitrogen deficiency in the microalgal growth medium leads to an increased carbohydrate content due to reduced protein synthesis; however, a lack of carbohydrate accumulation under this condition may also be due to hyper-accumulation of lipids [47]. In the present study, nitrogen depletion was linearly related to carbohydrate content (Figure 3). In particular, the carbohydrate content increased from 13.71% DW in the control medium to 28% DW in the nitrogen-free medium. DNA, chlorophyll, and proteins all contain nitrogen, and nitrogen limitation decreases microalgal growth and increases carbohydrate productivity [40]. In addition, reactive oxygen species (ROS) react with lipids, proteins, and DNA, leading to oxidative stress [17,48]. The most notable effect of nitrogen depletion is increased lipid accumulation, followed by increased chlorophyll decomposition and carbohydrate accumulation [49]. Although nitrogen deficiency leads to protein and chlorophyll decomposition, the carbohydrate content increased, which might be attributed to the increased storage of metabolites in the presence of sodium acetate as exo-organic carbon. Alternatively, nitrogen deficiency might lead to sugar condensation, which in turn increases the level of non- reduced sugars. Rehman and Anala found that nitrogen depletion increased the biosynthesis of lipids and starch in *Chlorococcum* sp. [50]. Other studies on microalgal growth under nitrogen starvation confirmed dramatic effects on photosynthesis, and this in turn led to changes in the lipids, carbohydrates, proteins, pigments, and fatty acid contents [51].

#### 2.2.3. Pigments

**Figure 4:**
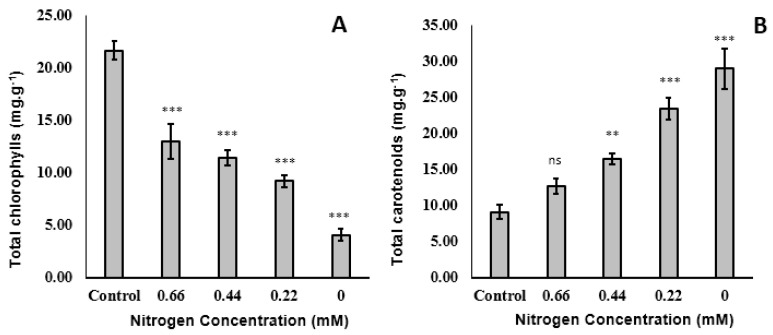
**Contents of** total chlorophyll (**A**) and carotenoids (**B**) in *Tisochrysis lutea* grown mixotrophically in different concentrations of NaNO_3_. ** *p* < 0.01, *** *p* < 0.001, ns: not significant with respect to the control (0.88 mM).

The effect of nitrogen depletion on chlorophyll content was similar to its effect on DW and protein content. In particular, the chlorophyll content decreased linearly with nitrogen level (Figure 4A). This was due to the close relationship between growth and chlorophyll content. The opposite effect was recorded regarding accumulation of carotenoids, in that nitrogen deficiency was linearly associated with increased carotenoid accumulation (Figure 4B). Other stressors, such as high salinity, excessive free radicals, and a high level of organic carbon, can also reduce chlorophyll and simultaneously enhance carotenoid accumulation. For instance, exposure of microalgal cells to low doses of cold plasma resulted in a significant increase of free radicals, which was accompanied by degradation of chlorophyll and reduction of growth, while a significant increase in carotenoids content was recorded in order to overcome the stress conditions [17]. The present study confirmed that the absence of nitrogen in the medium leads to DW and chlorophyll level reduction, but with a carotenoid content that was about 3-times greater than the control.

#### 2.2.4. Lipid Content

**Figure 5:**
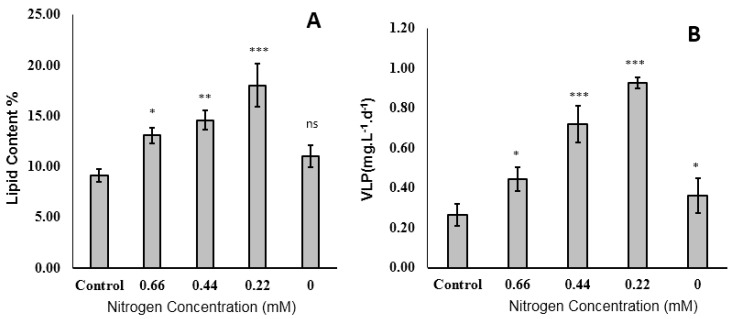
Lipid content (**A**) and volumetric lipid productivity (VLP) (**B**) of *Tisochrysis lutea* grown mixotrophically in different concentrations of NaNO_3._ * *p* < 0.05, ** *p* < 0.01, *** *p* < 0.001, ns: not significant with respect to the control (0.88 mM).

Previous studies confirmed that *Tisochrysis* sp. under optimized conditions can reach a specific growth rate up to 1.0 d^−1^, with lipid content of 20–30%, making this species a good candidate for a feedstock for industrial essential fatty acids and biodiesel production [52,53]. Under normal growth conditions, ω3 polyunsaturated fatty acids (PUFAs) are the most abundant fatty acids in *Tisochrysis*. Stressors, including nitrogen depletion, affect microalgal growth and markedly influence the lipid content and fatty acid profile. The maximum lipid content (18.04% DW) was recorded when these cells were grown in 0.22 mM NaNO_3_ (Figure 5A). De novo lipid synthesis is a response of algal cells during stress conditions by condensing two-carbon fragments to form fatty acids based on exo- or endo-organic carbon [8]. During stress conditions, chlorophyll is usually decomposed and algal cells obligatorily use the organic carbon to survive and store lipids resulting in the reduction of carbohydrates [17]. This explains the rise of oils and carotenes in the present study. In the presence of exo-organic carbon (sodium acetate in this study), cells utilize this molecule instead of activating catabolic pathways of cellular components. However, nitrogen-free cultures had a lipid content of 11.02% DW, which was not significantly different from the control. Under severe nitrogen depletion, a decrease in both dry weight and oil content was observed due to the utilization of lipids as a nutrient source to maintain cell survival, and at least 10% of the optimum nitrogen content was required to improve enzymatic processes and allow the survival of the cells [41]. Considering the growth together with lipid content, volumetric lipid productivity (VLP) was significantly increased due to reduction of nitrogen concentration in the medium to 0.22 mM (Figure 5B).

*T. lutea* may be considered a promising feedstock for biodiesel production because it accumulates about 20% of its DW as lipids, and this level is even greater under certain growth conditions [43]. Silitonga et al. reported *I. galbana* had a maximum lipid yield of only 8.41% DW [54]. Song et al. studied the growth of *I. galbana* in the presence of nitrogen depletion and concluded that increased glycolysis and citrate transport were mainly responsible for lipid anabolism in the presence of nitrogen limitation, and that the glyoxylate cycle, tricarboxylic acid cycle, and sulfur assimilation system were mainly responsible for lipid catabolism [46].

During the early stages of microalgal stress, there are increased levels of carotenoids, nutritional factors, lipids, and sugars, and decreased levels of proteins and chlorophyll [55,56]. Thus, photosynthesis markedly declines and DW remains relatively constant. The sugars produced by photosynthesis are responsible for osmotic regulation of the cells, so in the absence of photosynthesis, these cells can store carbon in different forms in order to avoid osmotic dysregulation. Because these alternative forms of carbon accumulate at much greater levels, the C/N ratio also increases. In addition, nitrogen deficiency often leads to the use of nicotinamide adenine dinucleotide phosphate (NADP) and adenosine triphosphate (ATP) (normally used for cellular growth) for biosynthesis of fatty acids resulting in lipid accumulation [57,58]. On the other hand, elevated nitrogen, phosphorous, and potassium levels had the same effect as nitrogen depletion in freshwater microalgae because they led to excessive salinity [59].

#### 2.2.5. Fatty Acids and Biodiesel Characteristics

The lipid composition and fatty acid methyl esters (FAME) profile are important determinants of biodiesel quality. The carbon chain length and extent of unsaturation are key parameters, as well as specific gravity, cetane number, iodine value, and higher heating value (HHV). The present results confirmed the production of 13 fatty acids in all treatments, and these fatty acids had 14 to 22 carbons (Table 2). For all tested nitrogen concentrations, myristic acid (C14:0) was the main saturated fatty acid (SFA), oleic acid (C18:1) was the main monounsaturated fatty acid (MUFA), and γ-linolenic acid (C18:3) was the main PUFA. However, nitrogen depletion had significant effects on the proportions of different fatty acids. Nitrogen deficiency in general increased unsaturation. In particular, in nitrogen-free medium, 39.53% of total fatty acids were PUFAs (2.2-fold greater than in the control medium [17.66%]) and MUFAs accounted for 31.45% of total fatty acids. Nitrogen depletion also led to a corresponding reduction in SFAs from 64.96% (control medium) to 29.02% (nitrogen-free medium). Previous studies also reported that nitrogen deficiency in the presence of organic carbon led to significant increases in the levels of PUFAs (mainly C16:3 and C18:3), and decreases of SFAs [60,61]. A microalgal FAME profile with a lower level of unsaturated fatty acids (especially four or fewer double-bonds) indicates better oxidation stability (Table 3) [24,62,63]. None of the studied treatments led to production of fatty acids with such high unsaturation.

The major biodiesel characteristics were also assessed when *T. lutea* was grown in different concentrations of nitrogen and our data compared with standard values in the U.S. (ASTM D2425, 2019) [64] and Europe (EN 12916, 2019) [65]. Generally, the values of specific gravity, kinematic viscosity, iodine value, and cetane number of all treatments were consistent with these recommended standards. However, nitrogen deficiency led to an increased HHV, which is advantageous from the perspective of energy efficiency. Thus, nitrogen limitation during the mixotrophic cultivation of *T. lutea* is a promising approach to achieve high lipid productivity and a FAME profile that is suitable for biodiesel production.

## 3. Materials and Methods

### 3.1. Microalgal Strain and Growth Conditions

*Tisochrysis lutea* (strain number CCAP 927/14) was provided by the Culture Collection of Algae and Protozoa (Oban, UK). The culture was maintained in a 50 mL vessel of F2 liquid medium [66].

The inoculum was prepared by culturing axenic cells in 150 mL of F2 medium until the culture reached the end of the exponential phase. Cells were then diluted with fresh medium to obtain the target inoculum concentration. The obtained inoculum was centrifuged and washed 3 times using acidified distilled water to remove all extracellular nutrients, and was then added to a 14 L transparent photobioreactor (2 m × 11 cm) that had different concentrations of NaNO_3_ (0.0, 0.22, 0.44, 0.66, and 0.88 mM). Mixotrophic growth was promoted by the addition of organic carbon (45 mM sodium acetate) and 6.0 ppm FeSO_4_.7H_2_O. Cultures were incubated under continuous light (120 µmol/m^2^/s) using an LED light system. Aeration was achieved using an airlift pump with a gentle oil-free air stream.

### 3.2. Growth Measurements

Biomass production was determined by measuring the dry weight at the end of each experiment. Samples (20 mL of culture) were centrifuged for 10 min at 3000× *g*, then the pellet was transferred to a pre-weighed dry Eppendorf tube. Samples were freeze-dried at −80 °C, and the cellular dry weight (DW) was obtained gravimetrically. Growth rate (GR) and the level of growth rate inhibition (I) were calculated according to the following equations;

GR (d^−1^) = (Lin2 − Lin1)/t (1)

I (%) = [(GR1 − GR2)/GR1] × 100
(2)

where Lin1 and Lin2 represent the initial dry weight and that after time (t) of incubation. GR1 and GR2 represent the growth rate of the control and the treatment, respectively.

### 3.3. Biochemical Analysis

At the end of each experiment, aeration was halted to allow overnight settling of cells. The upper aqueous phase was discarded, and the remaining concentrated culture was filtered, washed twice with distilled water, and then centrifuged for 10 min at 3000× *g*. Biomass was then subjected to biochemical analysis for determination of the levels of proteins, carbohydrates, lipids, pigments, moisture, and ash.

#### 3.3.1. Proteins and Carbohydrates

The crude protein content was determined as total nitrogen multiplied by 6.25, and soluble protein was determined following trichloroacetic acid (TCA) precipitation. The true protein content was estimated as the difference between crude and soluble protein [67]. The phenol sulfuric acid method [68] was used to determine the total carbohydrate content.

#### 3.3.2. Pigments

Pigment extraction was performed using 95% dimethyl sulfoxide DMSO [69]. The absorbance (*I*) was measured at wavelengths of 645 nm and 663 nm, then the concentrations of chlorophylls (as μg/g) were calculated according to Arnon [70]:

Chl-*a* = 12.7 (*I_663_*) − 2.69 (*I_645_*) (3)

Chl-*b* = 22.9 (*I_645_*) − 4.68 (*I_663_*) (4)

Total chlorophyll = 20.2 (*I_645_*) + 8.02 (*I_663_*) (5)

Total carotenoid content (as μg/g) was estimated by measuring the absorbance at 480, 663, and 645 nm and according to Kirk and Allen [71]:

Carotenoids = *I_480_* + (0.114 × *I_663_*) − (0.638 × *I_645_*) (6)

#### 3.3.3. Lipid Extraction and Determination

Biomass was frozen for 48 h, and then dried using vacuum filtration. The dry biomass was soaked for 24 h in the solvent (n-hexane–isopropanol, 3:2 *v*/*v*). After incubation, the extract was filtered, and the solvent was evaporated using a rotary evaporator. The lipid productivity (*VLP*, as mg L^−1^ d^−1^) was calculated according to Liu [72]:

*VLP* = *P_dwt_* × *L_c_* (7)

where *P_dwt_* is the sample DW and *L_c_* is the total lipid content at the harvest time.

Fatty acid methyl esters (FAMEs) were prepared and the fatty acid profile was determined as previously described [9]. Briefly, cells were collected from 15 mL of culture by centrifugation at 3000× *g*, then the pellet was heated in a boiling water bath for 3 min to deactivate intracellular lipases. After lipid extraction, lipid extracts were dried under a stream of argon, and 167 µL of 0.5 M sodium methoxide and 333 µL of 1:1 methanol:toluene were added and incubated at room temperature for 20 min. Afterwards, 50 µL of 37% HCl and 0.5 mL of 1 M NaCl were added, and FAMEs were extracted by 1.5 mL of hexane. After hexane evaporation, FAMEs were resuspended in 50 µL of acetonitrile. Gas chromatography (GC, Perkin Elmer Auto System XL) was used to measure FAMEs by injecting 1 µL. The oven temperature increased from 40 °C up to a final temperature of 260 °C at a rate of 4.0 °C/min, where it was held for 5 min. The characteristics of the different FAMEs were evaluated based on the fatty acid profile at different treatments [73], as previously described by Abomohra et al. [74].

### 3.4. Statistical Analysis

Experiments were carried out in three replicates, and the results are shown as the means ± standard errors (SEs). One-way analysis of variance (ANOVA) was applied to statistically analyze the data at different probability levels (*P*) using Statistica version 8.0 (StatSoft Inc., Tulsa, OK, USA).

## 4. Conclusions

The nitrogen concentration of the microalgal medium in the presence of sodium acetate as external carbon source showed a significant impact on the growth and lipid production of *T. lutea*. Although nitrogen deficiency inhibited growth, it stimulated the overall lipid productivity by modifying the metabolic processes toward lipid accumulation. However, severe nitrogen limitation resulted in a significant reduction in the lipid content. Thus, to ensure proper growth and lipid accumulation, severe nitrogen depletion is not advised. In addition, nitrogen deficiency enhanced the higher heating value of the resulting biodiesel, making the product more favorable for biodiesel production. Nitrogen limitation of *T. lutea* when grown under mixotrophic conditions produced abundant high-quality lipids, indicating this species has the potential to be used in large-scale biodiesel production. Further studies are required in order to evaluate the economic feasibility of this approach using *T. lutea*.

## Figures and Tables

**Table 1:** The impact of nitrogen concentration on the growth rate (GR) and the level of growth rate inhibition (I) in *Tisochrysis lutea*.

**NaNO_3_ (mM)**	0.88	0.66	0.44	0.22	0.0
**GR (d^−1^)**	0.187	0.171	0.149	0.101	0.059
**I (%)**	0.00	8.84	20.1	45.8	68.6

**Table 2:** Fatty acid profile of *Tisochrysis lutea* grown mixotrophically in different concentrations of NaNO_3_.

Fatty Acid	NaNO_3_ (mM)				
	0.88 (Control)	0.66	0.44	0.22	0
Myristic 14:0	25.75 ± 1.89	20.06 * ± 2.07	16.21 *** ± 1.00	10.27 *** ± 0.59	7.36 *** ± 0.58
Palmitic 16:0	17.26 ± 1.00	16.67 ± 1.06	14.99 ± 1.50	10.12 *** ± 0.99	9.47 *** ± 0.99
Stearic 18:0	12.04 ± 0.94	10.34 ± 0.82	8.92 ± 0.98	8.91 *** ± 0.59	8.17 *** ± 0.54
Arachidilic C20:0	9.91 ± 0.69	10.66 ± 1.35	8.24 ± 0.75	7.61 ± 0.15	4.02 *** ± 0.04
Myristoleic 14:1	3.11 ± 0.12	4.86 ± 0.80	6.14 * ± 0.59	8.14 *** ± 0.57	11.04 *** ± 1.28
Palmitoleic 16:1	6.13 ± 0.97	7.07 ± 0.78	7.89 ± 0.55	8.02 ± 0.18	8.74 * ± 0.50
Oleic 18:1	8.14 ± 0.55	8.45 ± 0.88	10.62 ± 1.94	10.67 ± 0.89	11.67 ± 0.88
Linoleic 18:2	2.10 ± 0.02	2.14 ± 0.12	3.08 * ± 0.12	8.14 *** ± 0.54	10.4 *** ± 0.18
α-Linolenic 18:3	2.01 ± 0.04	5.22 *** ± 0.05	6.14 *** ± 0.06	6.97 *** ± 0.67	7.12 *** ± 0.69
γ-Linolenic 18:3	2.84 ± 0.19	3.17 ± 0.31	4.37 ** ± 0.51	4.95 *** ± 0.13	5.17 *** ± 0.38
Arachidonic 20:4	5.69 ± 0.63	6.08 ± 0.16	6.13 ± 0.14	7.52 * ± 0.33	7.62 * ± 0.82
Eicosapentaenoic 20:5	2.94 ± 0.30	3.09 ± 0.46	3.49 * ± 0.22	3.51 * ± 0.25	4.13 ** ± 0.02
Docosahexaenoic 22:6	2.08 ± 0.22	2.19 ± 0.58	3.78 * ± 0.32	5.17 *** ± 0.93	5.09 *** ± 0.60
Saturated fatty acids SFA (%)	64.96 ± 4.52	57.73 * ± 5.30	48.36 *** ± 4.24	36.91 *** ± 2.33	29.02 *** ± 2.17
Monounsaturated fatty acids MUFA (%)	17.38 ± 1.64	20.38 ± 2.45	24.65 ** ± 3.07	26.83 ** ± 1.63	31.45 *** ± 2.66
Polyunsaturated fatty acids PUFA (%)	17.66 ± 1.40	21.89 ± 1.68	26.99 *** ± 1.36	36.26 *** ± 2.85	39.53 *** ± 2.68

* *p* < 0.05, ** *p* < 0.01, *** *p* < 0.001 with respect to the control (0.88 mM).

**Table 3:** Characteristics of biodiesels produced by *Tisochrysis lutea* grown mixotrophically in different concentrations of NaNO_3_.

Characteristics	NaNO_3_ (mM)					International Standards	
	0.88 (control)	0.66	0.44	0.22	0	U.S. (ASTM D2425)	Europe (EN 12916)
ADU	0.64	0.80	0.96	1.11	1.19	-	-
KV (mm^2^ s^−1^)	4.80	4.70	4.60	4.50	4.46	1.9–6.0	3.5–5.0
SG	0.88	0.88	0.88	0.88	0.88	0.85–0.9	-
CP (°C)	11.44	9.36	7.12	5.14	4.12	-	-
Cetane number	58.60	57.57	56.45	55.46	54.95	Min. 47	51–120
IV (g I_2_/100 g oil)	60.35	71.93	84.39	95.41	101.09	-	Max. 120
HHV (MJ kg^−1^)	39.66	39.94	40.23	40.49	40.63	-	-
Db ≥ 4 (wt%)	0.00	0.00	0.00	0.00	0.00	≤1	-

ADU, average degree of unsaturation; KV, kinematic viscosity; SG, specific gravity; CP, cloud point; IV, iodine value; HHV, higher heating value; Db, double bonds.
